# Impact of hospital care on incidence of bloodstream infection: the evaluation of processes and indicators in infection control study.

**DOI:** 10.3201/eid0702.010207

**Published:** 2001

**Authors:** S B Kritchevsky, B I Braun, E S Wong, S L Solomon, L Steele, C Richards, B P Simmons

**Affiliations:** University of Tennessee Health Sciences Center, Memphis, Tennessee, USA. skritchevsky@utmem.edu

## Abstract

The Evaluation of Processes and Indicators in Infection Control (EPIC) study assesses the relationship between hospital care and rates of central venous catheter-associated primary bacteremia in 54 intensive-care units (ICUs) in the United States and 14 other countries. Using ICU rather than the patient as the primary unit of statistical analysis permits evaluation of factors that vary at the ICU level. The design of EPIC can serve as a template for studies investigating the relationship between process and event rates across health-care institutions.


Special Issue

### Impact of Hospital Care on Incidence of Bloodstream Infection: The Evaluation of Processes and Indicators in Infection Control Study

**Stephen B. Kritchevsky,* Barbara I. Braun,† Edward S. Wong,‡ Steven L. Solomon,§ Lynn Steele,§ Cheryl Richards,† Bryan P. Simmons,¶* and the EPIC Study Group[1]**

*University of Tennessee Health Sciences Center, Memphis, Tennessee, USA; †Joint Commission on Accreditation of Healthcare Organizations, Oakbrook Terrace, Illinois, USA; ‡McGuire Veterans Administration Medical Center and Medical College of Virginia, Richmond, Virginia, USA; §Hospital Infections Program, Centers for Disease Control and Prevention, Atlanta, Georgia, USA; ¶Methodist Health Systems, Memphis, Tennessee, USA

The Evaluation of Processes and Indicators in Infection Control (EPIC) study assesses the relationship between hospital care and rates of central venous catheter-associated primary bacteremia in 54 intensive-care units (ICUs) in the United States and 14 other countries. Using ICU rather than the patient as the primary unit of statistical analysis permits evaluation of factors that vary at the ICU level. The design of EPIC can serve as a template for studies investigating the relationship between process and event rates across health-care institutions.

### Comparing Clinical Performance

Health-care organizations are increasingly expected to provide clinical outcomes data as measures of clinical quality to accrediting bodies, purchasers, and the public, under the premise that outcome variations indicate quality differences across organizations. Variation in clinical performance can result from variation in any number of factors, some relevant to improving the quality of care but many not. The best-studied source of variation in clinical performance measures is patient characteristics. Hospitals differ widely in the severity of illness and extent of coexisting illnesses in their patients, and much research has been devoted to developing risk adjustment methods to permit interhospital comparisons not confounded by patient characteristics (1). Hospitals also differ in methods of data abstraction and data management (2). Even subtle differences in definitions can introduce measurable variation in clinical performance(3).

Variations in patients, data collection, and definitions distract from collecting comparative data for quality improvement. To be useful, an indicator must be linked to variations in the processes of care provided since these processes are within the scope of control of the health-care organization. Furthermore, the "signal" must be separable from the "noise" of extraneous variation. Despite pressure to collect and disseminate clinical performance data as instruments of quality improvement, relatively little research has been done to establish their validity by demonstrating an association with process differences between hospitals.

In 1993, the Society for Healthcare Epidemiology of America (SHEA) responded to a growing concern among its membership about the sudden increase in the use of clinical performance comparisons to measure quality of health care. At the same time, the Joint Commission on Accreditation of Healthcare Organizations announced a plan to require all hospitals to collect an identical set of comparative indicators as part of its Agenda for Change Initiative. In 1994, the Joint Commission and SHEA formed a collaboration called the Project to Monitor Indicators (4) to foster the science of comparative indicators for the benefit of both organizations and the health-care community. The initial demonstration project, called the Comparison of Hospital Performance Indicators, was completed in 1997 (3). The second project, which is nearing completion, is called Evaluation of Processes and Indicators in Infection Control (EPIC). EPIC's area of

Address for correspondence: Stephen B. Kritchevsky, Department of Preventive Medicine, University of Tennessee, Memphis, 66 N. Pauline, Suite 633, Memphis, TN 38105; fax: 901-448-7641; e-mail: skritchevsky@utmem.edu

[1]Barbara I. Braun, Cheryl Richards, David Mitchell, Linda Matrician, Sandi Baus, Nina Mazzola, Robert Grisnak, Michael F. Parry, Diane Baranowsky, Merceditas S. Villanueva, Alice M. Stankus, Barbara Russell, Richard J. Duma, Jacquelyn Wolff, G. Merill Shore, Francis J.G. Liu, Mary J.K. Kim, Sharon Welbel, Mary Wisniewski, Stuart Johnson, Catherine O'Neill, Anna K. Huang, Ruth Carrico, Malkanthie I. McCormick, Kathryn Zink, Janice Piazza, Jacqueline Berry, Robert L. Pinsky, Jean Maurice, Terri Bethea, Christopher J. Sullivan, Barbara Bor, Joseph R. Thurn, James W. Lederer, Diann Allred, Jeffrey Engel, Sue Barnett, Peg Janasie, Martin Topiel, Carol Ward, Christine Filippone, Brian S. Koll, Prity L. Vaidya, Elizabeth DeHaan, Chatrchai Watanakunakorn, Mary Kundus, James E. Bross, Mary Dahlmann, Paul M. Newell, Ann Schlimm, Debra A. Runyan, Vicky Lieb, Joan Kies, Leonard Mermel, Steve Parent, Lynn Cromer, Bryan Simmons, Kelley Melton, Pam Falk, Gregory Bond, Jane M. Lane, Jacqueline P. Butler, Bonnie Greene, Edward Wong, Katharine Bryson, Mario Javier DeLuca, Cresio Romeu Pareira, Claudia Vallone Silva, Juan Menares, Jerome Robert, Stefan Weber, Marena Carlo, Kenji Kono, Najwa A. Khuri-Bulos, Chik Hyun Pai, ETM Smyth, Carol Jarvis, Ziad A. Memish, Marjeta Skerl, Antoni Trilla, Didier Pittet.

focus is bloodstream infections, specifically those in intensive-care unit (ICU) patients.

Because hospital epidemiology is a mature discipline, infection control indicators offer excellent opportunities to demonstrate how processes of care relate to infectious disease outcomes. Hospital epidemiology has long addressed surveillance techniques, disease definitions, patient risk factors, and process factors that may influence disease rates (5-7).

### EPIC Study Design

EPIC is two investigations under one name. The first investigation is designed to answer the following question: do the relative rankings of hospitals change, with indicators of bloodstream infection used for comparison? The design is relatively straightforward. With the assistance of the Centers for Disease Control and Prevention's Hospital Infections Program, the project identified six vendors offering different bloodstream infection indicators. A sample of 36 hospitals is collecting the data necessary to calculate these six indicators. When completed, the relative rankings of the hospitals across the set of indicators will be compared. The second investigation is designed to answer the following question: can variation in hospital care process explain variation in bloodstream infection rates across a sample of ICUs? The design for answering this question differs considerably from traditional epidemiologic designs (e.g., cohort and case-control designs).

### Patient Risk vs. Unit Rates

EPIC relates process performance to variation in bloodstream infection rates across ICUs. Traditional epidemiologic designs focus on the prediction of disease risk for the individual patient. In a traditional cohort study, the processes of care under scrutiny would be documented in ICU patients with central venous catheters. Primary bloodstream infections are relatively rare, even in this vulnerable population; however, this rarity presents practical problems in study design. Given an average 3% risk to each patient, prospective cohorts would have to include approximately 2,500 patients to have 80% power to detect as statistically significant a twofold relative risk associated with an exposure common to 25% of ICU patients. The case-control design was developed to address situations in which the outcome under study is uncommon; however, case-control studies establish exposure status after the disease has occurred. Therefore, not all varieties of exposure can be studied. In hospital epidemiology, exposures that are reliably documented in the medical record (coexisting diseases, for example) can be studied by a case-control approach. However, relevant aspects of the process of care are not always documented (e.g., the experience of the central venous catheter inserter or the number of attempts at insertion) and may be difficult to establish retrospectively.

Even if all relevant process factors could be documented in advance, some factors cannot be studied within a single ICU or even across a small number of ICUs. In many instances, process exposures are mandated by hospital, ICU, or infection control policy. In this situation, all patients within an ICU may have catheters inserted with specific types of barriers or have a similar skin preparation before catheter insertion. If there is no variation in the process under study within an ICU, that process cannot be evaluated by examining patients within that ICU. One would need to examine many ICUs with varied processes to relate the process to disease risk.

Ultimately, traditional designs cannot address the variation in unit rates because they focus on the wrong unit of analysis, i.e., the patient rather than the ICU. To study variation in ICU bloodstream infection rates, the ICU is the appropriate unit of analysis. The ICU rate is an aggregate measure that represents the average risk for bloodstream infection. Strong but infrequent determinants of patient risk have relatively little influence on the unit rate. A certain process factor, like gross contamination at the insertion site, may be related to a marked increase in bloodstream infection risk for individual patients but may occur so rarely that the overall rate of infection is not noticeably influenced. Even if a strong determinant of risk were relatively common, it would not necessarily be an important determinant of differences in bloodstream infection rates across ICUs. For an exposure to affect variation in rates between ICUs, two criteria must be met. First, the condition must be common enough to influence the bloodstream infection rate, i.e., it must have a fairly high attributable risk. Second, there must be variation between ICUs in the proportion of patients affected. Even a strong factor will not explain differences if every ICU has the same proportion of patients affected. Conversely, a relatively modest determinant of patient risk could account for a substantial proportion of the variation between ICU infection rates if ICUs varied greatly in the proportion of patients exposed. The average patient and average process determine the ICU infection rate since the ICU rate is a function of the average patient risk. The difference between individual risk and population rates has been extensively explored elsewhere (8).

When the ICU is the unit of analysis, important difficulties in evaluating process can be resolved. First, factors that vary at the level of the ICU can be studied appropriately. Factors not routinely charted can also be studied efficiently. Since the goal of the evaluation is to relate the average process to the ICU rate, only data sufficient to adequately characterize the average process are required. Therefore, every insertion in an ICU does not have to be followed; a random sample of insertions allows characterization of typical performance. On the other hand, many ICUs must be studied, since the sample size of the project is not the number of patients in ICUs but the number of ICUs being compared.

### EPIC Process Assessment Design

In 1998, the membership of SHEA and other interested persons were solicited to support participation of their respective hospitals in the study. Initially, 58 hospitals volunteered to participate (Table) (four were added later and eight withdrew). Data collection began in November 1998 and continued through January 2000, and data from 54 ICUs have been forwarded to the coordinating unit. The number of ICUs was determined by the willingness of epidemiologists and infection control personnel to participate in the study. However, the sample size is sufficient to evaluate important determinants of variation in ICU bloodstream infection rates. With a sample of 54 ICUs, a factor that explains 7% of the variance in the ICU rates would be statistically significant (alpha=0.05).

Because of its precise definitions and long history of use in the field, the National Nosocomial Infections Surveillance

Table. Hospitals participating in EPIC

| Characteristic | n | % |
|---|---|---|
| Major teaching hospital | 33 | 61.1 |
| NNIS participant | 18 | 33.3 |
| Location | | |
| United States* | 40 | 74.0 |
| International | 14 | 26.0 |
| ICU selected | | |
| Medical | 12 | 22.2 |
| Surgical | 4 | 7.4 |
| Medical/surgical | 34 | 63.0 |
| Other | 4 | 7.4 |
| Study ICU bed size | | |
| 1-9 | 12 | 22.2 |
| 10-14 | 24 | 44.4 |
| 15-19 | 8 | 14.8 |
| ≥20 | 10 | 18.5 |

(NNIS) System's central venous catheter-associated primary bloodstream infection indicator in ICU patients was used (9). To establish the rate, each ICU reported all qualifying infections to the coordinating unit throughout the study period. Units also reported their central-line days throughout the study period. Using these data elements, the coordinating unit calculated the NNIS indicator rate for each hospital.

Data on process and patient characteristics were collected for a random sample of central venous catheter insertions in patients admitted to the study ICUs. All hospitals were provided with the same list of five randomly selected dates and times each month. The study volunteers identified the first catheter insertion occurring after each random date and time and recorded a number of patient and process factors and interviewed the line inserter to document details of the insertion. Interviews were conducted within 48 hours of the insertion. It was not necessary for the insertion to have occurred in the study ICU; any patient who was admitted to the ICU within 8 hours of central venous catheter insertion qualified. Up to 65 insertions were documented during the study in each ICU. Each patient was monitored for bloodstream infection for 2 days after discharge from the ICU.

The higher the number of insertions assessed, the more precise the assessment of process. However, the increase in precision with sample size is not linear. The increase in precision in the estimate of the mean is a function of the standard error, which in turn is a function of the inverse of the square root of the sample size. Therefore, the return from increasing the sample size by a given amount decreases as the sample size increases. For example, adding 45 new observations to an initial sample of 20 observations increases the relative precision in the estimate of the mean by approximately 80%. Adding 45 new observations to an initial sample of 55 increases the precision only by approximately 30%. The value of 65 was selected because it was large enough to provide acceptably precise performance estimates but was not so large as to preclude voluntary participation in the study.

Data elements collected in EPIC are as follows: 1) Factors related to the patient: age, sex, primary and secondary diagnoses, length of ICU stay, dialysis, neutropenia, active treatment for cancer involving either chemotherapy or radiotherapy, albumin <3 g/L, burns involving >10% of body surface area, HIV/AIDS, current immunosuppressive therapy, and surgery under general anesthesia within 2 weeks before insertion. 2) Factors related to the line: type of central line, number of lumens, coating with antimicrobial material, anatomic site of insertion, location of insertion, urgency of insertion, use of the line for hyperalimentation, line exchange over a guide wire, and duration of the line. 3) Factors related to the insertion of the line: use of barrier precautions (sterile gown, mask, large drape, small drape), type of dressing applied, time from initial needlestick until line secured, number of sites attempted before completion, number of attempts made at the final insertion site, experience of the inserter (years inserting and number of lines inserted in the past 6 months), professional background of the inserter, and unusual occurrences during the insertion. 4) Factors related to the organization: number and kinds of ICUs within the hospital, presence of an infection control committee, length of time tracking bloodstream infection rates, experience tracking central line-days, NNIS participation, number of blood cultures done in the previous year, staffing for ICU surveillance, percentage of lines managed by a team, percentage of lines using a needleless systems, and number of in-service training sessions provided to the ICU staff in the previous 6 months. 5) Factors related to the study ICU: number of hours devoted to surveillance in the study ICU, experience and training of the infection control staff doing surveillance, total of registered nurse hours in the ICU, number of agency nurse hours used for staffing, number of "float" nurse hours used for staffing, total number of patient days, and minimum experience required for a new ICU nurse.

### Conclusions

The goal of comparative measurement for quality improvement is to identify opportunities for improvement by showing which organizations have superior processes. However, a clear link between process and indicator needs to be established before the indicator can be confidently used for this purpose. The design of EPIC provides an opportunity to relate the typical care process directly to bloodstream infection rates in ICUs. Because the ICU is the unit of analysis, EPIC can evaluate process factors that could not be addressed by studies within a single ICU, specifically processes and policies that apply to all patients within an ICU. In addition, because the sample of patients in each ICU are followed for the development of bloodstream infections, the study affords a unique opportunity to compare an analysis based on patient risk with one based on unit rates.

The coordinating activities of EPIC are supported by a cooperative agreement with CDC's Hospital Infections Program under the Prevention Epicenters Program.

Dr. Kritchevsky is associate professor and director and chair of the masters of epidemiology program, Department of Preventive Medicine, University of Tennessee. He also chairs the Society of Healthcare Epidemiology of America's Project to Monitor Indicators, under which the EPIC study was conducted.

### References

1. Iezzoni LI, Ash AS, Shwartz M, Daley J, Hughes JS, Mackiernan YD. Judging hospitals by severity-adjusted mortality rates: the influence of the severity-adjustment method. Am J Public Health 1996;86:1379-87.
2. Romano PS, Mark DH. Bias in coding of hospital discharge data and its implications for quality assessment. Med Care 1994;32:81-90.

3. Kritchevsky SB, Braun BI, Gross PA, Newcomb CS, Kelleher CA, Simmons BP. Definition and adjustment of cesarean section rates and assessments of hospital performance. Int J Quality Health Care 1999;11:283-91.
4. Kritchevsky SB, Simmons BP, Braun BI. The Project to Monitor Indicators: a collaborative effort between the Joint Commission on Accreditation of Healthcare Organizations and the Society for Healthcare Epidemiology of America. Infect Control Hosp Epidemiol 1995;16:33-5.
5. Pearson ML, Hospital Infection Control Practices Advisory Committee. Guideline for prevention of intravascular device-related infections. Infect Control Hosp Epidemiol 1996;17:438-73.
6. Farr BM. Nococomial infections related to use of intravascular devices inserted for short-term vascular access. In: Mayhall CG, editor. Hospital epidemiology and infection control. 2nd ed. Philadelphia: Lippincott Williams & Wilkins; 1999. p. 157-64.
7. Mermel LA. Prevention of intravascular catheter-related infections. Ann Intern Med 2000;132:391-402.
8. Rose G. The strategy of preventive medicine. Oxford: Oxford University Press; 1992.
9. Emori TG, Culver DH, Horan TC, Jarvis WR, White JW, Olson DR, et al. National Nosocomial Infections Surveillance System (NNIS): description of surveillance methods. Am J Infect Control 1991;19:19-35.
